# Associations between Oxidative/Nitrosative Stress and Thyroid Hormones in Pregnant Women—Tainan Birth Cohort Study (TBCS)

**DOI:** 10.3390/antiox11020334

**Published:** 2022-02-09

**Authors:** Po-Keng Cheng, Hsin-Chang Chen, Pao-Lin Kuo, Jung-Wei Chang, Wan-Ting Chang, Po-Chin Huang

**Affiliations:** 1 National Institute of Environmental Health Sciences, National Health Research Institutes, Miaoli 35053, Taiwan; ansd39@nhri.edu.tw (P.-K.C.); wtchang2@nhri.edu.tw (W.-T.C.); 2 Department of Chemistry, Tunghai University, Taichung 40704, Taiwan; hsinchang@ntu.edu.tw; 3 Department of Obstetrics and Gynecology, National Cheng Kung University Hospital, College of Medicine, Tainan 70101, Taiwan; paolink@mail.ncku.edu.tw; 4 Institute of Environmental and Occupational Health Sciences, School of Medicine, National Yang Ming Chiao Tung University, Taipei 11221, Taiwan; jungwei723@ym.edu.tw; 5 Research Center for Environmental Medicine, Kaohsiung Medical University, Kaohsiung 80756, Taiwan; 6 Department of Medical Research, China Medical University Hospital, China Medical University, Taichung 40402, Taiwan

**Keywords:** oxidative stress, nitrosative stress, thyroid hormone, lipid peroxidation, pregnancy

## Abstract

Oxidative and nitrosative stress have been linked to thyroid function in both animal and human studies. In the present study, the associations between oxidative and nitrosative stress and thyroid hormones were investigated. Measurements were obtained from 97 Taiwanese pregnant women at the first, second, and third trimesters. Levels of five oxidative and nitrosative stress biomarkers (8-hydroxy-2′-deoxyguanosine [8-OHdG], 8-nitroguanine [8-NO_2_Gua], 4-hydroxy-2-nonenal-mercapturic acid [HNE-MA], 8-isoprostaglandin F2α [8-isoPGF_2α_], and malondialdehyde [MDA]) were measured using urine samples, and levels of five thyroid hormones (triiodothyronine [T_3_], thyroxine [T_4_], free T_4_, thyroid-stimulating hormone [TSH], and T_4_-binding globulin [TBG]) were measured in blood samples. Multiple linear regressions and linear mixed-model regressions were conducted to determine the associations between oxidative or nitrosative stress biomarkers and thyroid hormones in pregnant women. We found that TSH was negatively and significantly associated with 8-NO_2_Gua (−14%, 95% CI [−26.9% to −1.1%]) and HNE-MA (−23%, 95% CI [−35.9% to −10.0%]) levels. However, T_4_ (3%, 95% CI [0.2%–5.8%]) and free T_4_ (4.3%, 95% CI [0.8%–7.8%]) levels were positively and significantly associated with 8-NO_2_Gua. The T_4_ to TBG and free T_4_ to TBG ratios were positively and significantly associated with 8-NO_2_Gua level (T_4_/TBG: 3.6%, 95% CI [0.5%–6.7%]; free T_4_/TBG: 5.6%, 95% CI [0.2%–11.1%]). However, the TSH to T_4_ ratio was negatively and significantly associated with 8-NO_2_Gua level (−17.3%, 95% CI [−30.4% to −4.3%]). The T_3_ to TSH ratio was positively and significantly associated with HNE-MA level (25.2%, 95% CI [11.2%–39.2%]). However, the TSH to T_4_ and TSH to free T_4_ ratios were negatively and significantly associated with HNE-MA level (TSH/T_4_: −21.2%, 95% CI [−34.5% to −7.8%] and TSH/free T_4_: −24.0%, 95% CI [−38.3% to −9.6%]). Our findings suggest that an imbalance of oxidative and nitrosative stress may alter thyroid hormone homeostasis during pregnancy. Disruption of the maternal thyroid homeostasis during pregnancy would affect embryonic and fetal development.

## 1. Introduction

Oxidative and nitrosative stress are defined as an imbalance between the synthesis of prooxidant substances and antioxidant defenses. The most important prooxidants are reactive oxygen species (ROS) and reactive nitrogen species (RNS). ROS and RNS are key pathogenetic factors that play roles in a wide range of illnesses. ROS and RNS are highly chemically reactive molecules due to their single unpaired electrons in their outer orbitals. Therefore, ROS free radicals, such as superoxide anions and hydroxyls, play a unique pathogenetic role [1]. Oxidative stress is associated with a variety of chronic diseases, including cardiovascular disorders [2].

The thyroid hormones, namely triiodothyronine (T_3_) and thyroxine (T_4_), are key regulators of growth, development, and metabolism that are produced and released by the thyroid gland. Thyroid hormones control cell proliferation and the basal metabolic rate by altering metabolic and energy homeostasis, thermogenesis, and the transcription of genes that regulate cell proliferation [3,4]. Thus, thyroid hormones play a role in a variety of physiological activities, including energy metabolism, central nervous system growth and formation, tissue differentiation, and reproduction [5].

Thyroid hormones exert a major influence on oxidative stress because of their function in cellular metabolism and oxygen consumption. The negative feedback mechanisms in the endocrine system regulate thyroid mechanisms to maintain them at appropriate levels. If any changes were to occur in thyroid hormone levels, such changes may alter the redox environment by altering the amount and activity of mitochondrial respiratory chain components, leading to an increase in ROS generation, which are generally limited by antioxidants. An overproduction of ROS induces thyroid hormones to consume more oxygen, causing oxidative stress and damage to cellular structures, lipids, and proteins [6].

Some studies have suggested that maternal or fetal oxidative stress plays a key role in the pathophysiology of low birth weight [7,8,9]. For example, Matsubasa et al. reported that as birth weights at sampling increased, the average levels of urinary 8-hydroxy-2′-deoxyguanosine (8-OHdG) decreased (infants under 1000 g with 8-OHdG levels 29.5 ± 16.4 μmol/mol creatinine, 1000–1500 g with 23.8 ± 14.9, over 1500 g with 16.1 ± 8.5, and control with 10.9 ± 7.2) [9]. Kim et al. reported that the concentrations of maternal urinary 8-OHdG and malondialdehyde (MDA) were inversely associated with the birth weight of full-term deliveries after controlling for potential confounders [10]. Therefore, monitoring oxidative stress in pregnant women is key in uncovering the link between oxidative stress and pregnancy outcomes [10]. Furthermore, maintaining maternal thyroid homeostasis is essential for fetal growth and development during pregnancy [11]. Hyperthyroidism and hypothyroidism in pregnant women have been associated with adverse birth outcomes, such as preterm births and low birth weights [12,13]. Minor changes in thyroid function in pregnant women might negatively affect fetal health [14,15]. However, a few studies have assessed the impact of oxidative and nitrosative stress.

In the present study, we aimed to explore the relationships between oxidative or nitrosative stress and thyroid hormone in pregnant Taiwanese women. The hypothesis is that an imbalance of oxidative and nitrosative stress would alter thyroid hormone homeostasis at different trimesters during pregnancy. We investigate the associations that oxidative or nitrosative stress has on thyroid hormone levels through an exploration of the effects of lipid peroxidation (4-hydroxy-2-nonenal-mercapturic acid [HNE-MA], 8-isoprostaglandin F2α [8-isoPGF2_α_], and MDA) and oxidative (8-OHdG) and nitrosative (8-nitroguanine [8-NO_2_Gua]) DNA damage on the thyroid functions of Taiwanese pregnant women at their first, second, and third trimester visits through repeated measurements analysis [1,16]. The chosen biomarkers were based on relevant studies [1,10,16,17,18,19,20], in which some chosen oxidative and nitrosative stress biomarkers were linked with birth outcomes and different endocrine-disrupting chemicals (EDCs), such as phenols and phthalates. Meanwhile, exposure window of different trimesters may vary and induce different levels of oxidative and nitrosative stress.

## 2. Materials and Methods

### 2.1. Participants and Study Design

The data for this study were obtained from the Tainan Birth Cohort Study (TBCS 2013–14) project. Our data collection process has been detailed in our prior publications [20,21,22]. In brief, our study included pregnant women whose blood biochemical examination (alpha fetal protein and free-hCG) were abnormal, or whose advanced maternal age (>35 years) indicated the suggestion for amniocentesis, as recommended by gynecologists at the National Cheng Kung University Hospital. Pregnant women with preeclampsia, abnormal chromosomal disease, gestational diabetes mellitus, intrauterine growth restriction, or other complications were excluded. A total of 97 pregnant women with normal-DNA infant were included for analysis. At the initial study visit (median: 18 gestational weeks), the participants provided sociodemographic information (age, education, occupational history, and social economic status.), pregnancy history (gestational age, menarche age, and parity), lifestyle habits (tobacco use, passive smoking, and alcohol consumption), and exposure history (exposure to di-(2-ethylhexyl) phthalate [DEHP]-contaminated products before the DEHP episode and nutritional supplement consumption). Urine and blood samples of pregnant women were collected at three visits at median gestation times of 18, 26, and 39 weeks.

The study protocol was approved by the Research Ethics Committee of the National Health Research Institutes (No. EC1020302) at 23 September 2013 and the Institutional Review Board of National Cheng Kung University Hospital (No. A-ER-102-104) on 27 June 2013 in Taiwan. Informed consent was obtained from each participant before study enrollment.

### 2.2. Analytical Method for Oxidative and Nitrosative Stress Biomarkers

Using an isotope dilution liquid chromatography–tandem mass spectrometry (LC–MS/MS), we evaluated the levels of four oxidative and nitrosative stress biomarkers, 8-OHdG, 8-NO_2_Gua, 8-IsoPGF_2α_, and HNE-MA per a method in the literature [16,20]. The levels of MDA in urine samples were assessed by measuring the level of thiobarbituric acid (TBA) reactive substances, which result from the reaction that occurs between MDA and TBA at high temperatures ranging from 90 to 100 °C. A commercial kit (Cayman Chemicals No.10009055, Ann Arbor, MI, USA) was used to analyze urine samples according to the manufacturer’s instructions. At 530–540 nm, the formed MDA–TBA product was colorimetrically measured. The intra-assay and inter-assay coefficients of variation were 7.6% and 5.1%, respectively [23].

### 2.3. Analytical Method for Measuring Serum Thyroid Hormones and Urinary Creatinine

Serum thyroid function and urinary creatinine concentrations were measured by a Taiwan Accreditation Foundation–certified laboratory (Nos. 1447 and 1673), which has been recognized by the International Laboratory Accreditation Cooperation Mutual Recognition Arrangement [24]. For creatinine, combined clinical chemistry and immunoassay tests (Modular Analytics Serum Work Area; Roche Diagnostics) were employed to analyze urine samples (which had been stored at −20 °C within one week before analysis). Thyroid function was assessed using an electrochemiluminescence immunoassay (Elecsys 2010 and Modular Analytics E170; Roche Diagnostics) to measure the serum concentrations of T_4_, free T_4_, T_3_, thyroid-stimulating hormone (TSH), and thyroxine-binding globulin (TBG). All analyses were conducted by a blinded technician and in a random order.

### 2.4. Statistical Analysis

The characteristics of the participants were described in terms of the mean and standard deviation for the continuous variables of age and gestational week at enrollment and in terms of percentage for education level, household income, primipara, folic acid consumption, alcohol consumption, and tobacco exposure. For each visit, detection rates, geometrical means (GMs), and medians were used to describe the distributions of oxidative or nitrosative stress biomarkers and thyroid hormones. To analyze the trend across each visit, linear mixed models with random intercepts for study participants and study visit as an independent variable were used to compare GMs between the three visits [18]. Spearman’s correlation coefficients were used to assess the correlations between oxidative and nitrosative stress biomarkers and thyroid hormones. To investigate intraindividual variability in measurements across study visits, intraclass correlation coefficients (ICCs) for oxidative and nitrosative stress biomarkers and thyroid hormones were calculated. The natural logarithm (ln) of the levels of oxidative and nitrosative stress biomarkers and thyroid hormones were used to meet the normality assumption. Multiple linear regressions were fitted to investigate the associations between oxidative and nitrosative stress biomarkers and thyroid hormones. The associations between the intertertile increase in oxidative or nitrosative stress biomarkers and the percentage of change in thyroid hormones across visits were then assessed using linear mixed models with first-order autoregressive covariance structures and random intercepts for participants, in which concentration variables (i.e., oxidative and nitrosative stress biomarkers) were classified into tertiles (i.e., 0–2) and enter as continuous variables into regression. Selection of covariates was based on the literature review, availability, and their statistical significance in the models. In order to separate any impact of creatinine on the associations, urinary creatinine as a covariate has been suggested as an alternative to creatinine-corrected analytes [25]. Finally, we chose the continuous covariates of age and urinary creatinine level for adjusting. All statistical analyses were conducted in R version 4.1.0 (R Foundation for Statistical Computing, Vienna, Austria).

## 3. Results

### 3.1. Demographic Characteristics of Participants

At the time of enrollment, the 97 participating pregnant women had a mean gestation time of 18.3 ± 1.3 weeks and an average age of 35.1 ± 3.5 years. Primiparas accounted for approximately half of the participants (43.3%). The majority (95.9%) had a university-level education, and 82.1% were from households with an annual income more than NTD 500,000 (approximately USD 15,625). Over half of the participants (55.4%) had consumed folic acid throughout the month preceding the start of the study. Few reported having drunk alcohol (1.0%) or smoked (2.1%); however, 16.5% reported secondhand smoke exposure (Table 1).

### 3.2. Distributions of Oxidative and Nitrosative Stress Biomarkers and Thyroid Hormones

The distributions of oxidative and nitrosative stress biomarkers and serum thyroid hormone levels are summarized in Table 2. With the exception of 8-isoPGF_2α_, which had a detection rate ranging from 61% to 77% across the three study visits, nearly all the oxidative and nitrosative stress biomarkers and thyroid hormones had detection rates of 100%.

No significant differences were noted between each of these oxidative and nitrosative stress biomarkers and thyroid hormones across the three visits. However, a higher TSH level was noted at the third visit (GM = 2.4 μIU/mL) than those noted at the first and second visits (GM = 1.2 and 1.1 μIU/mL, respectively), and a lower HNE-MA level was noted at the third visit (GM = 26.9 ng/mL) than those noted at the first and second visits (GM = 52.9 and 54.3 ng/mL, respectively). The GM ranges across the three study visits were observed as follows 105.6–122.3 ng/dL for T_3_, 8.7–8.9 μg/dL for T_4_, 0.6–0.8 ng/dL for free T_4_, 34.7–38.3 μg/mL for TBG, 8.2–10.5 μmole/L for MDA, 2.6–2.9 ng/mL for 8-OHdG, 1.5–1.6 ng/mL for 8-NO_2_Gua, and 0.6–0.9 ng/mL for 8-isoPGF_2α_. The ICCs for thyroid hormones ranged from 8.4% (free T_4_) to 63.1% (TBG), and the ICCs for oxidative and nitrosative stress biomarkers ranged from −2.8% (8-isoPGF_2α_) to 18.8% (8-OHdG). Table 3 presents distribution of thyroid hormone ratios in pregnant women by study visits.

We ran Mann–Whitney U test and Kruskal–Wallis tests to compare oxidative/nitrosative stress and thyroid hormone levels in classified groups based on potential confounding factors listed in Table 1, but the results were not significant (Supplementary Table S1). We also compared the thyroid hormone levels of participants against a reference value for pregnant women in each trimester. For TSH, 26% of our participants had higher TSH levels than the reference value for pregnant women in the third trimester. For T3, 52% and 74% of our participants had lower T3 levels than the reference values for pregnant women in the second and third trimesters, respectively. For T4, 29% and 28% of our participants had higher T4 levels than the reference values for pregnant women in the first and third trimesters, respectively. For free T4, 54% and 40% of our participants had lower free T4 levels than the reference values for pregnant women in the first and second trimesters, respectively. For TBG, 67%, 41% and 40% of our participants had higher TBG levels than the reference values for pregnant women in the first, second, and third trimesters, respectively (Supplementary Table S2).

### 3.3. Correlations between Oxidative and Nitrosative Stress Biomarkers and Thyroid Hormones

**Figure 1:**
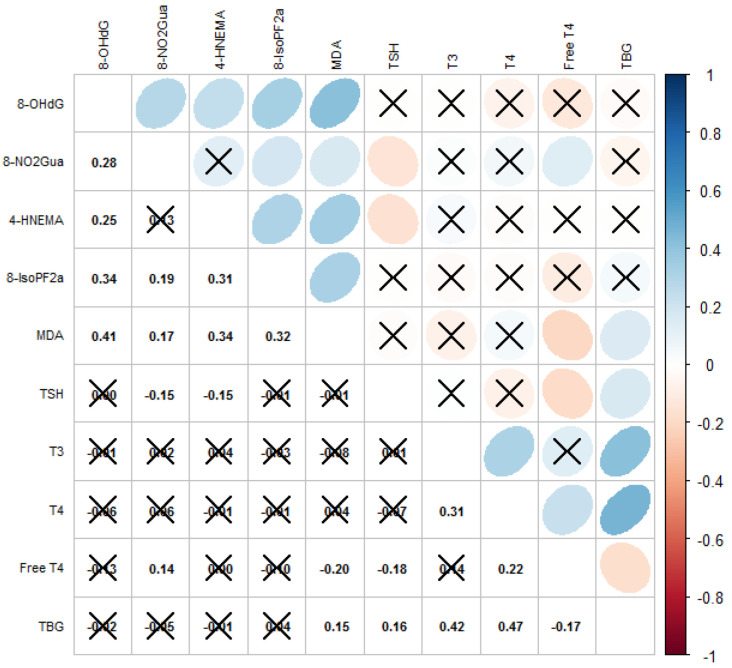
Correlations among oxidative/nitrosative stress biomarkers and thyroid hormones. Cross mark means correlation is not significant (*p*-value ≥ 0.05); blue means positively correlated; red means negatively correlated.

The correlation matrix with the Spearman’s coefficients of the oxidative and nitrosative stress biomarkers and thyroid hormones is displayed in Figure 1. Levels of most biomarkers of oxidative or nitrosative stress as well as thyroid hormones were moderately correlated (Spearman’s ρ = −0.18–0.47). We observed that the level of TSH was significantly correlated with levels of 8-NO_2_Gua (Spearman’s ρ = −0.15) and HNE-MA (Spearman’s ρ = −0.16), that the level of free T_4_ was significantly correlated with the levels of 8-NO_2_Gua (Spearman’s ρ = 0.14) and MDA (Spearman’s ρ = −0.20), and that the level of TBG was significantly correlated with the level of MDA (Spearman’s ρ = 0.15).

### 3.4. Associations between Oxidative and Nitrosative Stress Biomarkers and Thyroid Hormones in Multiple Linear Regression and Linear Mixed Models

**Figure 2:**
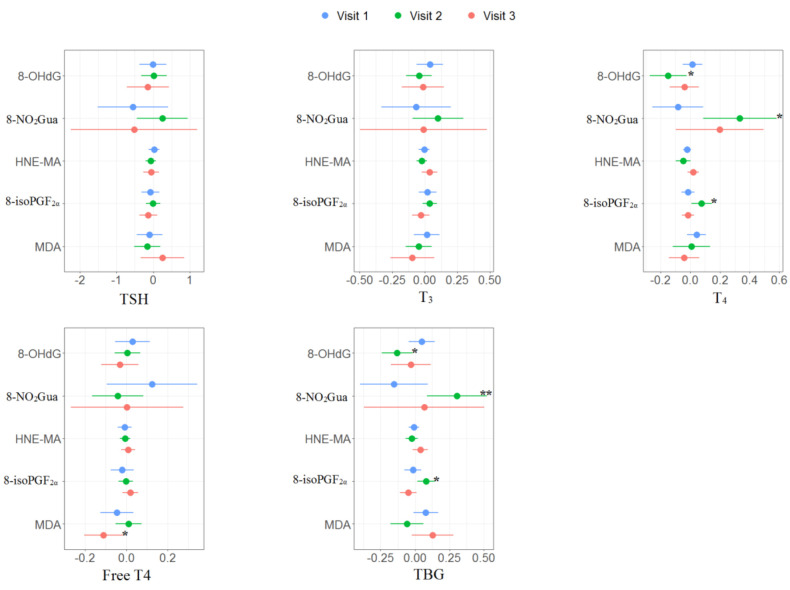
Unit change in oxidative/nitrosative stress biomarkers concentrations in association with concentrations of thyroid hormones across three visits. Multiple linear regressions are adjusted for age and creatinine; * *p* < 0.05, ** *p* < 0.01, *** *p* < 0.001.

The multiple linear regression results of the association between oxidative or nitrosative stress biomarkers and thyroid hormones for each trimester are presented in Supplementary Tables S3–S5 and Figure 2. No significant association was found in the first trimester. For the second trimester, 8-OHdG was negatively associated with T_4_ (β: −0.15, 95% CI [−0.28 to −0.02]) and TBG (β: −0.13, 95% CI [−0.25 to −0.02]); 8-NO_2_Gua was positively associated with T_4_ (β: 0.33, 95% CI [0.08 to 0.59]) and TBG (β: 0.30, 95% CI [0.08 to 0.53]); 8-isoPGF_2α_ was positively associated with T_4_ (β: 0.08, 95% CI [0.01 to 0.15]) and TBG (β: 0.08, 95% CI [0.01 to 0.15]). For the third trimester, MDA was negatively associated with free T_4_ (β: −0.11, 95% CI [−0.21 to −0.01]).

**Figure 3:**
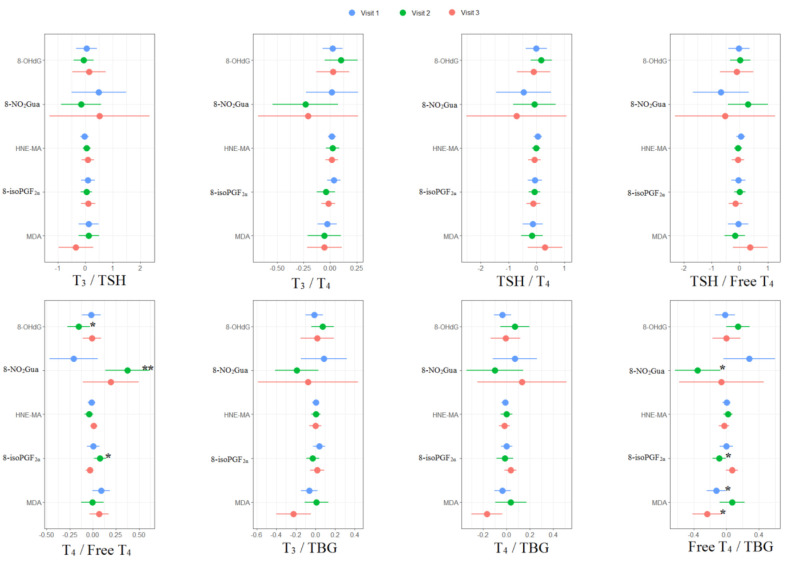
Unit change in oxidative/nitrosative stress biomarker concentrations in association with concentrations of thyroid hormone ratios across three visits. Multiple linear regressions are adjusted for age and creatinine; * *p* < 0.05, ** *p* < 0.01, *** *p* < 0.001.

For the associations between thyroid hormone ratios and oxidative or nitrosative stress biomarkers, MDA was negatively associated with free T_4_ to TBG ratio (β: −0.12, 95% CI [−0.24 to −0.01]) in the first trimester. For the second trimester, 8-OHdG was negatively associated with T_4_ to free T_4_ ratio (β: −0.15, 95% CI [−0.28 to −0.03]); 8-NO_2_Gua was positively associated with T_4_ to free T_4_ ratio (β: 0.37, 95% CI [0.13 to 0.62]) but negatively associated with free T_4_ to TBG ratio (β: −0.35, 95% CI [−0.64 to −0.07]); 8-isoPGF_2α_ was positively associated with T_4_ to free T_4_ ratio (β: 0.08, 95% CI [0.01 to 0.15]) but negatively associated with free T_4_ to TBG ratio (β: −0.09, 95% CI [−0.18 to −0.01]). For the third trimester, MDA was negatively associated with free T_4_ to TBG ratio (β: −0.24, 95% CI [−0.42 to −0.05]) (Supplementary Tables S3–S5 and Figure 3).

**Figure 4:**
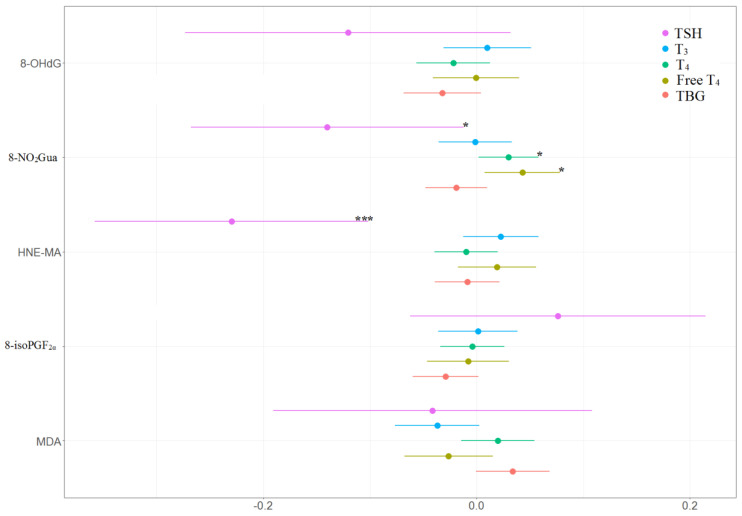
Percent changes in concentrations of thyroid hormones in association with tertile increase in oxidative/nitrosative stress biomarkers concentrations across three visits. Linear mixed models with random intercept were adjusted for age and creatinine; * *p* < 0.05, ** *p* < 0.01, *** *p* < 0.001.

**Figure 5:**
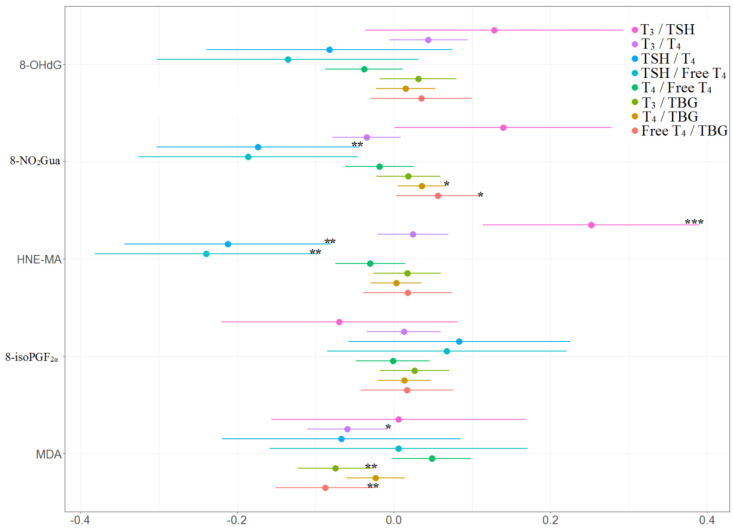
Percent changes in concentrations of thyroid hormone ratios in association with tertile increase in oxidative/nitrosative stress biomarkers concentrations across three visits. Linear mixed models with random intercept were adjusted for age and creatinine; * *p* < 0.05, ** *p* < 0.01, *** *p* < 0.001.

The associations between the percentage of change in concentrations of thyroid hormones and the tertile increases in oxidative or nitrosative stress biomarker concentrations across three visits are presented in Table 4 and Figure 4 and Figure 5. TSH level was negatively and significantly associated with 8-NO_2_Gua level (−14%, 95% CI [−26.9% to −1.1%]) and HNE-MA level (−23%, 95% CI [−35.9% to −10.0%]). T_4_ level was only positively and significantly associated with 8-NO_2_Gua level (3%, 95% CI [0.2%–5.8%]), and free T_4_ was only positively and significantly associated with 8-NO_2_Gua (4.3%, 95% CI [0.8%–7.8%]). The T_4_ to TBG and free T_4_ to TBG ratios were positively and significantly associated with 8-NO_2_Gua level (T_4_/TBG: 3.6%, 95% CI [0.5%–6.7%]; free T_4_/TBG: 5.6%, 95% CI [0.2%–11.1%]). However, the TSH to T_4_ ratio was negatively and significantly associated with 8-NO_2_Gua level (−17.3%, 95% CI [−30.4% to −4.3%]). The T_3_ to TSH ratio was positively and significantly associated with HNE-MA level (25.2%, 95% CI [11.2%–39.2%]). However, the TSH to T_4_ and TSH to free T_4_ ratios were negatively and significantly associated with HNE-MA level (TSH/T_4_: −21.2%, 95% CI [−34.5% to −7.8%]; TSH/free T_4_: −24.0%, 95% CI [−38.3% to −9.6%]). The T_3_ to T_4_, T_3_ to TBG, and free T_4_ to TBG ratios were negatively and significantly associated with MDA level (T_3_/T_4_: −5.9%, 95% CI [−11.0% to −0.8%]; T_3_/TBG: −7.5%, 95% CI [−12.3% to −2.6%]; free T_4_/TBG: −8.8%, 95% CI [−15.1% to −2.4%]).

## 4. Discussion

To our knowledge, this is the first study of pregnant Taiwanese women to investigate the relationships between oxidative and nitrosative stress and thyroid hormones by using repeated measurements analysis. In our study, urinary levels of 8-isoPGF_2α_, 8-OHdG, HNE-MA, and 8-NO_2_Gua remained mostly stable across three visits (Table 2), falling within the ranges reported for Taiwanese pregnant women in the third trimester [28] and those reported for the general healthy population [16]. The levels of 8-isoPGF2α and MDA reported for pregnant women in the United States [29] and Korea [10] were similar to our measurements. Nitrosative stress, measured using 8-NO_2_Gua, was positively associated with T_4_ and free T_4_ levels but negatively associated with TSH level. Lipid peroxidation, measured using HNE-MA, was negatively associated with TSH level. These indicated that the nitrosative stress and lipid peroxidation may alter thyroid hormone homeostasis during pregnancy. In general Taiwanese adults, Huang et al. observed negative associations between 8-NO_2_Gua and T_4_ levels and between MDA and T_4_ levels; they also observed positive associations between 8-NO_2_Gua and free T_4_ levels, and between MDA/8-OHdG and free T_4_ levels [23]. Zhang et al. reported negative associations between 8-isoPGF_2α_ and free T_3_/free T_4_ levels for patients with benign thyroid nodules, and they reported a positive association between 8-isoPGF_2α_ and free T_4_ levels among patients with malignant thyroid nodules [30]. However, we did not observe any significant association between 8-OHdG or 8-isoPGF_2α_ and thyroid hormones in our study. Variations of the associations between oxidative or nitrosative stress and thyroid hormone levels could be due to differences in study design and participants.

In this study, we investigated the impacts of oxidative or nitrosative stress on thyroid hormones; however, thyroid hormones could also cause oxidative stress in target tissues. Thyroid hormones have long been known to induce lipid oxidative damage. Studies have demonstrated that mice are less susceptible to thyroid hormone-induced lipid oxidative damage. In addition, hyperthyroidism was demonstrated to increase protein oxidation in rat livers and testes, as indicated by higher protein-bound carbonyl concentration [31]. Enhanced oxidative stress involving enzymatic and nonenzymatic antioxidants is associated with both hyperthyroidism and hypothyroidism [32,33]. Furthermore, some complications from hyperthyroidism are specifically related to oxidative stress in target tissues [33,34]. The interactions between oxidative stress and thyroid hormones are complicated because oxidative stress could also be induced by thyroid hormones.

An excess of TSH has been linked to the development of oxidative stress in patients with thyroid carcinoma [1,35]. Other studies have observed lipid peroxidation in both overt and subclinical hypothyroidism, as indicated by an increase in MDA level [1,36]. However, in the present study, the increase in nitrosative stress (measured using 8-NO_2_Gua) was related to a decrease in TSH and increase in T_4_/free T_4_ levels; the increase in lipid peroxidation (measured using HNE-MA) was related to a decrease in TSH. Such opposite findings could result from pregnancy, in which raised oxidative stress with consequent enhanced lipid peroxidation as the TSH levels decreased would lower the risk of miscarriage. In addition, the increase in nitrosative stress (measured using 8-NO_2_Gua) was related to a decrease in the TSH to T_4_ ratio and increases in the T_4_ to TBG and in the free T_4_ to TBG ratios; an increase in lipid peroxidation (measured using HNE-MA) was related to an increase in the T_3_ to TSH ratio and decreases in the TSH to T_4_ as well as the TSH to free T_4_ ratios; the increase in lipid peroxidation (measured using MDA) was related to decreases in the T_3_ to T_4_, T_3_ to TBG, and free T_4_ to TBG ratios. These indicated that nitrosative stress and lipid peroxidation would alter the thyroid hormone homeostasis during pregnancy.

Because higher metabolic turnover and tissue oxygen consumption occur during pregnancy, it is physiologically linked to increased oxidative stress and reduced antioxidant capacity [37,38]. Antioxidant upregulation may be generated by environmental exposure-induced lipid peroxidation [1], which may contribute to the counteracting effects of oxidative stress on thyroid hormone transfer and function in the placenta. Placental oxidative stress plays a key role in the pathophysiology of placental-related disorders that put fetal development at risk [39]. However, the implication and relevance of oxidative stress in thyroid function and thyroid hormone balance require further clarification [40].

Normal fetal growth and development, particularly early fetal neurodevelopment, require thyroid homeostasis during pregnancy [11]. The mother is the main source of thyroid hormones for the fetus in early pregnancy (before 20 gestational weeks), while fetal thyroid function begins later in pregnancy (after 20 gestational weeks), although maternal thyroid hormones are still relatively essential [41]. Higher maternal free T4 levels in the first trimester of pregnancy have been linked to lower birth weight and an increased risk of small for gestational age [42]. In the present study, the increase in nitrosative stress (measured using 8-NO_2_Gua) was related to increase in T_4_ and free T_4_ levels, which indicated that an imbalance of oxidative and nitrosative stress could alter thyroid hormone homeostasis during pregnancy and affect fetal health. The treatment of hyper or hypothyroidism during pregnancy was debated. Thus, to avoid potential consequences, mitigation of possible risk factors are crucial, i.e., consumption of natural antioxidants would be recommended.

Huang et al. [23] and Zhang et al. [30] found that nitrosative DNA damage and lipid peroxidation may play mediating roles in the effects of phthalate exposure on thyroid hormones in humans. Yao et al. [40] reported a mediating role of oxidative stress biomarkers in the associations between organophosphate esters exposure and thyroid hormones in a population of pregnant women. Waits et al. [20] demonstrated that exposure to phthalates in pregnant women, particularly later in pregnancy, may lead to oxidative and nitrosative stress through DNA damage and lipid peroxidation. Thyroid homeostasis disruption caused by phthalate exposure may contribute to oxidative stress because thyroid hormones may also function as oxidants and cause DNA damage, most likely through phenolic groups [1,20,43]. Furthermore, environmental exposure may influence thyroid hormone levels through induced oxidative and nitrosative stress.

Ours is the first study on oxidative and nitrosative stress biomarkers and thyroid hormones in pregnant Taiwanese women; in addition, we included five different biomarkers for lipid peroxidation and oxidative and nitrosative DNA damage, some of which have not been previously assessed in relation to thyroid hormones. Another strength of our study was the use of repeated measurements analysis, which resulted in more robust estimations of the associations between oxidative and nitrosative stress and thyroid hormones during pregnancy. However, the small sample size of our study limits the generalizability of our findings. Because we considered only a few covariates, unmeasured confounding likely occurred. Furthermore, because intraindividual variability in oxidative and nitrosative stress biomarkers remains undetermined [44], the single spot urine sampling of our investigation is an additional limitation.

## 5. Conclusions

Our findings suggest that an imbalance of oxidative and nitrosative stress may alter thyroid hormone homeostasis during pregnancy. Disruption of the maternal thyroid homeostasis during pregnancy would affect embryonic and fetal development. To verify these associations, further mechanistic, prospective, and large-scale epidemiological studies are required.

## Figures and Tables

**Table 1:** Demographic characteristics of participated Taiwan’s pregnant women (N = 97).

Characteristics	Mean ± SD
Age (years)	35.09 ± 3.51
Gestational week at enrollment (weeks)	18.25 ± 1.34
	**n (%)**
Education	
High school (or earlier) (≤12 years)	4 (4.1)
University (12–16 years)	76 (78.4)
Postgraduate (≥16 years)	17 (17.5)
Annual household income (USD) ^a^	
<15,625	17 (17.9)
15,625–31,250	50 (52.6)
≥31,250	28 (29.5)
Primiparas	42 (43.3)
Folic acid consumption ^b^	36 (55.4)
Active cigarette smoking before pregnancy ^c^	2 (2.1)
Passive cigarette smoking before pregnancy ^d^	16 (16.5)
Alcohol consumption before pregnancy ^e^	1 (1.0)

^a^ Two missing values in annual household income; currency exchange rate of USD to new Taiwan dollar (TWD) is 1:32. ^b^ Consumed folic acid during the past month; 32 missing values in folic acid consumption. ^c^ Consumed at least one cigarette per day. ^d^ People around are often cigarette smoking. ^e^ Consumed at least 100 mL of alcohol per week; 1 missing value in alcohol consumption before pregnancy.

**Table 2:** Distribution of oxidative/nitrosative stress biomarkers, thyroid hormones, and creatinine in pregnant women by study visits (N = 218) ^a^.

Parameters	Visit 1 (*n* = 97)			Visit 2 (*n* = 63)			Visit 3 (*n* = 58)			P _trend_ ^b^	ICC
	DR ^a^	GM	Median (95% CI)	DR	GM	Median (95% CI)	DR	GM	Median (95% CI)		%
Oxidative/nitrosative stress											
8-OHdG (ng/mL)	100	2.6	2.1 (1.8–2.6)	100	2.9	2.3 (2.2–3.3)	100	2.6	2.6 (2.1–2.9)	0.818	18.8
8-NO_2_Gua (ng/mL)	100	1.6	1.5 (1.4–1.6)	100	1.5	1.4 (1.3–1.5)	100	1.5 ^c^	1.4 (1.4–1.5)	0.043 *	2.0
HNE-MA (ng/mL)	100	52.9	59.6 (40.1–81.2)	100	54.3	57.7 (29.3–90.5)	100	26.9 ^c^	30.5 (17.7–42.3)	0.010 *	7.5
8-IsoPGF2_α_ (ng/mL)	64	0.6	0.6 (0.5–0.8)	61	0.7	0.8 (0.5–1.0)	77	0.9 ^c^	1.0 (0.8–1.3)	0.022 *	–2.8
MDA (μmole/L)	100	8.2	7.8 (6.6–9.0)	100	10.5 ^c^	10.6 (8.2–12.5)	100	8.8	9.3 (7.4–11.8)	0.296	11.0
Thyroid hormones											
TSH (μIU/mL) ^d^	100	1.0	1.2 (1.0–1.4)	100	1.1	1.1 (1.0–1.3)	100	2.4 ^c^	2.9 (2.3–3.2)	<0.001 ***	30.2
T_3_ (ng/dL) ^d^	100	122.3	126 (118–130)	100	116.2 ^c^	115.0 (108.0–122.0)	100	105.6 ^c^	110.0 (97.0–117.0)	<0.001 ***	45.9
T_4_ (μg/dL) ^d^	100	8.9	8.8 (8.5–9.2)	100	8.7	8.9 (8.6–9.4)	100	8.7	8.6 (8.3–9.2)	0.313	44.5
Free T_4_ (ng/dL) ^d^	100	0.8	0.8 (0.7–0.9)	100	0.6 ^c^	0.6 (0.6–0.7)	100	0.6 ^c^	0.6 (0.6–0.6)	<0.001 ***	8.4
TBG (μg/mL) ^d^	100	34.7	36.2 (33.7–37.8)	100	37.8 ^c^	39.4 (38.4–43.4)	100	38.3 ^c^	40.3 (38.7–42.6)	<0.001 ***	63.1
Creatinine (mg/dL)	99	49.8	49.9 (40.0–61.0)	100	63.5	66.0 (44.5–85.7)	100	73.9 ^c^	81.4 (64.7–101.8)	0.008 **	29.8

^a^ DR(%)–detection rate; ICC–intraclass correlation coefficient; GM–geometric mean; 8-OHdG–8-hydroxy-2′-deoxyguanosine; 8-NO_2_Gua–8-nitroguanine; HNE-MA–4-hydroxy-2-nonenal-mercapturic acid; 8-IsoPGF2_α_–8-isoprostaglandin F_2α_; MDA–malondialdehyde; TSH–thyroid-stimulating hormone; T_3_–triiodothyronine; T_4_–thyroxine; TBG –T_4_-binding globulin. ^b^ Trend in geometrical means across the three visits; *: *p* < 0.05; **: *p* < 0.01; ***: *p* < 0.001. ^c^ Geometrical mean is significantly (*p* < 0.05) different from the first visit. ^d^ The laboratory reference ranges of adults were TSH (0.35–4.94 μIU/mL), T_3_ (58–159 ng/dL), T_4_ (4.87–11.72 μg/dL), free T_4_ (0.70–1.48 ng/dL), and TBG (15.8–25.4 μg/mL). The reference ranges for pregnant women were TSH (0.1–4.0 μIU/mL, first trimester; 0.2–4.0 μIU/mL, second trimester; 0.3–4.0 μIU/mL, third trimester), T_3_ (97–149 ng/dL, first trimester; 117–169 ng/dL, second trimester; 123–162 ng/dL, third trimester), T_4_ (6.5–10.1 μg/dL, first trimester; 7.5–10.3 μg/dL, second trimester; 6.3–9.7 μg/dL, third trimester), free T_4_ (0.8–1.2 ng/dL, first trimester; 0.6–1.0 ng/dL, second trimester; 0.5–0.8 ng/dL, third trimester), and TBG (18–32 μg/mL, first trimester; 28–40 μg/mL, second trimester; 26–42 μg/mL, third trimester). [26,27].

**Table 3:** Distribution of thyroid hormone ratios in pregnant women by study visits (N =218) ^a^.

Parameters	Visit 1 (*n* = 97)		Visit 2 (*n* = 63)		Visit 3 (*n* = 58)		P _trend_ ^b^	ICC
	GM	Median (95% CI)	GM	Median (95% CI)	GM	Median (95% CI)		(%)
T_3_/TSH	123.7	96.1 (85.8–127.5)	108.7 ^c^	104.3 (80.7–122.5)	44.7 ^c^	39.5 (36.0–44.0)	<0.001 ***	4.4
T_3_/T_4_	13.7	13.6 (12.9–14.3)	13.4	12.9 (11.8–14.6)	12.1 ^c^	12.1 (11.2–12.7)	0.001 **	5.5
TSH/T_4_	0.1	0.1 (0.1–0.1)	0.1 ^c^	0.1 (0.1–0.1)	0.3 ^c^	0.3 (0.3–0.4)	<0.001 ***	35.7
TSH/Free T_4_	1.2	1.4 (1.3–1.6)	1.7 ^c^	1.8 (1.6–2.0)	3.7 ^c^	4.3 (3.2–4.9)	<0.001 ***	25.4
T_4_/Free T_4_	11.0	10.7 (10.2–11.8)	13.8 ^c^	14.3 (13.5–14.9)	13.7 ^c^	13.6 (13.2–14.5)	<0.001 ***	18.4
T_3_/TBG	3.5	3.6 (3.2–3.8)	3.1 ^c^	3.0 (2.8–3.3)	2.8 ^c^	2.8 (2.5–2.9)	<0.001 ***	27.9
T_4_/TBG	0.3	0.3 (0.2–0.3)	0.2 ^c^	0.2 (0.2–0.2)	0.2 ^c^	0.2 (0.2–0.2)	<0.001 ***	47.0
Free T_4_/TBG	0.02	0.02 (0.02–0.02)	0.02 ^c^	0.02 (0.01–0.02)	0.02 ^c^	0.02 (0.01–0.02)	<0.001 ***	40.3

^a^ ICC–intraclass correlation coefficient; GM–geometric mean. ^b^ Trend in geometrical means across the three visits; *: *p* < 0.05; **: *p* < 0.01; ***: *p* < 0.001. ^c^ Geometrical mean is significantly (*p* < 0.05) different from the first visit.

**Table 4:** Percent changes ^a^ in concentrations of thyroid hormones in association with tertile increase in oxidative/nitrosative stress biomarker concentrations across three visits (N = 218).

Thyroid Hormones	8-OHdG	8-NO_2_Gua	HNE-MA	8-isoPGF_2α_	MDA
	Δ%(95%CI)	Δ%(95%CI)	Δ%(95%CI)	Δ%(95%CI)	Δ%(95%CI)
**TSH**	−12.1 (−27.5, 3.4)	−14 (−26.9, −1.1) *	−23.0 (−35.9, −10.0) ***	7.6 (−6.4, 21.6)	−4.1 (−19.1, 10.8)
**T_3_**	1.0 (−3.1, 5.1)	−0.1 (−3.6, 3.3)	2.3 (−1.3, 5.8)	0.1 (−3.6, 3.9)	−3.7 (−7.7, 0.3)
**T_4_**	−2.2 (−5.7, 1.3)	3.0 (0.2, 5.8) *	−0.1 (−4.0, 2.0)	−0.4 (−3.4, 2.7)	2.0 (−1.5, 5.5)
**Free T_4_**	−0.04 (−4.1, 4.0)	4.3 (0.8, 7.8) *	1.9 (−1.8, 5.6)	−0.8 (−4.6, 3.0)	−2.6 (−6.8, 1.6)
**TBG**	−3.2 (−6.9, 0.5)	−1.9 (−4.8, 1.0)	−0.9 (−3.9, 2.2)	−2.9 (−6.0, 0.2)	3.4 (−0.1, 6.9)
**T_3_/TSH**	12.8 (−3.7, 29.3)	13.9 (0.1, 27.8)	25.2 (11.2, 39.2) ***	−6.9 (−22.0, 8.1)	0.6 (−15.8, 17.0)
**T_3_/T_4_**	4.4 (−0.7, 9.5)	−3.5 (−7.9, 0.9)	2.5 (−2.1, 7.0)	1.3 (−3.5, 6.1)	−5.9 (−11.0, −0.8) *
**TSH/T_4_**	−8.2 (−24.1, 7.6)	−17.3 (−30.4, −4.3) **	−21.2 (−34.5, −7.8) **	8.4 (−5.8, 22.5)	−6.7 (−21.9, 8.5)
**TSH/Free T_4_**	−13.5 (−30.2, 3.2)	−18.6 (−32.6, −4.6)	−24.0 (−38.3, −9.6) **	6.8 (−8.7, 22.2)	0.6 (−15.8, 17.1)
**T_4_/Free T_4_**	−3.8 (−8.8, 1.2)	−1.8 (−6.2, 2.5)	−3.0 (−7.5, 1.5)	−0.1 (−4.9, 4.7)	4.9 (−0.2, 10.0)
**T_3_/TBG**	3.1 (−1.8, 8.0)	1.9 (−2.2, 6.0)	1.7 (−2.6, 6.0)	2.7 (−1.8, 7.1)	−7.5 (−12.3, −2.6) **
**T_4_/TBG**	1.5 (−2.3, 5.4)	3.6 (0.5, 6.7) *	0.3 (−3.0, 3.6)	1.3 (−2.0, 4.8)	−2.3 (−6.0, 1.5)
**Free T_4_/TBG**	3.5 (−3.0, 10.1)	5.6 (0.2, 11.1) *	1.8 (−3.9, 7.5)	1.7 (−4.3, 7.7)	−8.8 (−15.1, −2.4) **

^a^ Linear mixed models with random intercept were adjusted for age and creatinine; * *p* < 0.05, ** *p* < 0.01, *** *p* < 0.001.

## Data Availability

The data presented in this study are available in this manuscript and supplementary material.

## Supplementary Materials

The following supporting information can be downloaded at: https://www.mdpi.com/article/10.3390/antiox11020334/s1, Table S1: Potential confounding factors check, Table S2: Proportions (%) of thyroid hormones outside reference ranges for pregnant women, Table S3: Multiple linear regression in concentrations of thyroid hormones in association with unit change in oxidative/ nitrosative stress biomarkers concentrations in visit 1 (N = 97), Table S4: Multiple linear regression in concentrations of thyroid hormones in association with unit change in oxidative/ nitrosative stress biomarkers concentrations in visit 2 (N = 63), Table S5: Multiple linear regression in concentrations of thyroid hormones in association with unit change in oxidative/ nitrosative stress biomarkers concentrations in visit 3 (N = 58).
