# Supplementary material for: Associations between Oxidative/Nitrosative Stress and Thyroid Hormones in Pregnant Women—Tainan Birth Cohort Study (TBCS)
Source: Antioxidants (Basel). 2022 Feb 9;11(2):334. doi: 10.3390/antiox11020334 (PMC8868566; doi:10.3390/antiox11020334)
Supplement: Supplementary file 1 [file antioxidants-11-00334-s001.zip › antioxidants-1545909-supplementary.pdf]

Supplementary information.

Table S1. Potential confounding factors check.

Factors	TSH	T_3	T_4	Free T_4	TBG	8-OHdG	8-NO_2Gua	HNE-MA	8-IsoPGF2_α	MDA
**Age** (Pearson correlation)										
Visit 1	0.027	**0.207**	0.078	0.115	0.166	0.074	-0.079	-0.017	0.140	0.086
Visit 2	0.111	0.169	0.050	-0.161	**0.277**	-0.178	-0.146	0.038	-0.018	0.092
Visit 3	0.121	0.054	**0.280**	0.157	**0.263**	-0.122	-0.085	0.159	-0.027	-0.007
**Education** (Kruskal–Wallis test *p*-value)										
Visit 1	0.162	**0.012**	0.369	**0.023**	0.067	0.408	0.420	0.682	0.766	0.731
Visit 2	0.364	0.321	0.249	0.327	0.099	0.669	0.570	0.763	0.566	0.159
Visit 3	0.966	0.991	0.666	0.225	0.373	0.251	0.187	0.635	0.603	0.383
**Annual household income** (Kruskal–Wallis test *p*-value)										
Visit 1	0.341	0.237	0.638	0.260	0.122	0.273	0.381	0.188	0.171	0.070
Visit 2	0.168	0.375	0.128	0.459	0.755	0.567	0.875	0.573	0.234	0.907
Visit 3	0.955	0.966	0.507	0.750	0.178	0.199	0.072	0.172	0.464	0.884
**Primiparas** (Mann–Whitney U test *p*-value)										
Visit 1	0.710	0.819	0.546	**0.040**	0.833	0.267	0.965	0.305	0.903	0.659
Visit 2	0.457	**0.039**	0.651	0.051	0.638	0.854	0.519	0.795	0.200	0.113
Visit 3	**0.012**	0.683	0.276	0.188	0.491	0.340	0.962	0.781	0.207	0.506
**Folic acid consumption** (Mann–Whitney U test *p*-value)										
Visit 1	0.273	0.436	0.300	**0.034**	0.480	**0.042**	**0.012**	0.435	0.296	0.557
Visit 2	0.410	0.894	0.069	0.477	0.448	0.319	0.587	0.801	0.232	**0.045**
Visit 3	0.339	0.963	0.121	0.646	0.127	0.511	0.664	0.871	0.672	0.212
**Active cigarette smoking before pregnancy** (Mann–Whitney U test *p*-value)										
Visit 1	0.183	0.228	0.869	0.182	0.751	**0.019**	0.576	0.191	0.064	0.073
Visit 2	0.178	0.601	0.284	0.457	0.216	0.260	0.601	0.161	0.697	0.527
Visit 3	0.232	0.905	0.858	0.135	0.256	0.591	0.199	0.720	0.632	0.244
**Passive cigarette smoking before pregnancy** (Mann–Whitney U test *p*-value)										
Visit 1	0.676	0.648	0.586	**0.014**	0.973	0.655	**0.044**	0.411	0.431	0.384
Visit 2	0.271	0.229	0.255	0.160	0.279	0.323	0.535	0.771	0.668	0.658
Visit 3	0.134	0.668	0.492	0.565	0.417	0.082	0.910	0.373	0.901	0.259
**Alcohol consumption before pregnancy** (Mann–Whitney U test *p*-value)										
Visit 1	0.248	0.407	0.626	0.129	0.396	0.330	0.207	0.801	0.324	0.899
Visit 2	0.503	0.118	0.241	0.170	0.361	0.402	0.696	0.241	0.108	0.889
Visit 3	N.A. (because all participants in visit 3 didn't drink alcohol)									

Table S2. Proportions (%) of thyroid hormones outside reference ranges for pregnant women in this study.

	TSH	T_3	T_4	Free T_4	TBG
**Outside reference range**					
1st trimester	5.15	30.93	31.96	53.61	68.04
2nd trimester	4.76	57.14	36.51	39.68	53.57
3rd trimester	32.76	77.59	27.59	10.34	51.72
**Lower than reference range**					
1st trimester	3.09	13.40	3.09	53.61	1.03
2nd trimester	3.17	52.38	17.46	39.68	12.50
3rd trimester	6.90	74.14	0	3.45	12.07
**Higher than reference range**					
1st trimester	2.06	17.53	28.87	0	67.01
2nd trimester	1.59	4.76	19.05	0	41.07
3rd trimester	25.86	3.45	27.59	6.89	39.65

The reference ranges for pregnant women were TSH (0.1-4.0 μIU/mL, first trimester; 0.2-4.0 μIU/mL, second trimester; 0.3-4.0 μIU/mL, third trimester), T_3 (97-149 ng/dL, first trimester; 117-169 ng/dL, second trimester; 123-162 ng/dL, third trimester), T_4 (6.5-10.1 μg/dL, first trimester; 7.5-10.3 μg/dL, second trimester; 6.3-9.7 μg/dL, third trimester), free T_4 (0.8-1.2 ng/dL, first trimester; 0.6-1.0 ng/dL, second trimester; 0.5-0.8 ng/dL, third trimester), and TBG (18-32 μg/mL, first trimester; 28-40 μg/mL, second trimester; 26-42 μg/mL, third trimester). [26, 27]

Table S3. Multiple linear regression [a] in concentrations of thyroid hormones in association with unit change in oxidative/ nitrosative stress biomarkers concentrations in visit 1 (N=97).

Thyroid hormones/ oxidative/ nitrosative stress biomarkers	8-OHdG	8-NO_2Gua	HNE-MA	8-isoPGF$_{2\alpha}$	MDA
	β (95%CI)	β (95%CI)	β (95%CI)	β (95%CI)	β (95%CI)
**TSH**	-0.001 (-0.37, 0.37)	-0.55 (-1.53, 0.42)	0.02 (-0.13, 0.17)	-0.07 (-0.32, 0.17)	-0.10 (-0.46, 0.26)
**T_3**	0.04 (-0.07, 0.14)	-0.07 (-0.34, 0.20)	-0.01 (-0.05, 0.04)	0.02 (-0.05, 0.09)	0.02 (-0.08, 0.11)
**T_4**	0.01 (-0.05, 0.08)	-0.08 (-0.26, 0.09)	-0.02 (-0.05, 0.00)	-0.02 (-0.06, 0.03)	0.04 (-0.02, 0.11)
**Free T_4**	0.03 (-0.05, 0.12)	0.13 (-0.10, 0.35)	-0.01 (-0.04, 0.03)	-0.02 (-0.08, 0.04)	-0.05 (-0.13, 0.03)
**TBG**	0.05 (-0.05, 0.14)	-0.15 (-0.40, 0.10)	-0.01 (-0.05, 0.03)	-0.02 (-0.08, 0.05)	0.08 (-0.01, 0.17)
**T_3 / TSH**	0.04 (-0.35, 0.43)	0.49 (-0.53, 1.50)	-0.03 (-0.19, 0.13)	0.10 (-0.16, 0.35)	0.11 (-0.26, 0.49)
**T_3 / T_4**	0.02 (-0.07, 0.12)	0.02 (-0.23, 0.27)	0.02 (-0.02, 0.05)	0.04 (-0.03, 0.10)	-0.03 (-0.12, 0.06)
**TSH / T_4**	-0.02 (-0.40, 0.37)	-0.47 (-1.47, 0.53)	0.05 (-0.11, 0.20)	-0.06 (-0.31, 0.20)	-0.14 (-0.51, 0.22)
**TSH / Free T_4**	-0.03 (-0.42, 0.35)	-0.68 (-1.69, 0.33)	0.03 (-0.12, 0.19)	-0.06 (-0.31, 0.20)	-0.05 (-0.42, 0.32)
**T_4 / Free T_4**	-0.02 (-0.12, 0.09)	-0.21 (-0.47, 0.06)	-0.01 (-0.05, 0.03)	0.004 (-0.06, 0.07)	0.09 (-0.01, 0.19)
**T_3 / TBG**	-0.01 (-0.10, 0.08)	0.09 (-0.15, 0.32)	0.003 (-0.03, 0.04)	0.04 (-0.02, 0.10)	-0.06 (-0.15, 0.02)
**T_4 / TBG**	-0.04 (-0.11, 0.04)	0.07 (-0.12, 0.26)	-0.01 (-0.04, 0.02)	-0.000 (-0.05, 0.05)	-0.04 (-0.11, 0.04)
**Free T_4 / TBG**	-0.02 (-0.14, 0.11)	0.28 (-0.05, 0.60)	0.000 (-0.05, 0.05)	-0.004 (-0.09, 0.08)	**-0.12 (-0.24, -0.01)** *

[a] adjusted for age and creatinine; * $p < 0.05$, ** $p < 0.01$, *** $p < 0.001$.

Table S4. Multiple linear regression [a] in concentrations of thyroid hormones in association with unit change in oxidative/ nitrosative stress biomarkers concentrations in visit 2 (N=63).

Thyroid hormones/ oxidative/ nitrosative stress biomarkers	8-OHdG	8-NO_2Gua	HNE-MA	8-isoPGF$_{2\alpha}$	MDA
	β (95%CI)	β (95%CI)	β (95%CI)	β (95%CI)	β (95%CI)
**TSH**	0.02 (-0.34, 0.38)	0.25 (-0.46, 0.96)	-0.07 (-0.21, 0.07)	-0.004 (-0.20, 0.19)	-0.17 (-0.53, 0.20)
**T_3**	-0.04 (-0.15, 0.06)	0.10 (-0.10, 0.30)	-0.02 (-0.06, 0.02)	0.04 (-0.02, 0.09)	-0.05 (-0.15, 0.06)
**T_4**	**-0.15 (-0.28, -0.02)** *	**0.33 (0.08, 0.59)** *	-0.05 (-0.10, 0.00)	**0.08 (0.01, 0.15)** *	0.01 (-0.12, 0.14)
**Free T_4**	0.01 (-0.06, 0.07)	-0.04 (-0.17, 0.09)	-0.01 (-0.03, 0.02)	-0.002 (-0.04, 0.03)	0.01 (-0.05, 0.08)
**TBG**	**-0.13 (-0.25, -0.02)** *	**0.30 (0.08, 0.53)** **	-0.02 (-0.07, 0.02)	**0.08 (0.01, 0.15)** *	-0.06 (-0.18, 0.06)
**T_3 / TSH**	-0.06 (-0.44, 0.31)	-0.15 (-0.90, 0.59)	0.04 (-0.10, 0.19)	0.04 (-0.17, 0.25)	0.12 (-0.26, 0.50)
**T_3 / T_4**	0.10 (-0.05, 0.26)	-0.23 (-0.55, 0.08)	0.02 (-0.04, 0.09)	-0.04 (-0.13, 0.05)	-0.05 (-0.22, 0.11)
**TSH / T_4**	0.17 (-0.23, 0.56)	-0.08 (-0.86, 0.70)	-0.02 (-0.18, 0.13)	-0.08 (-0.30, 0.14)	-0.17 (-0.57, 0.22)
**TSH / Free T_4**	0.01 (-0.36, 0.38)	0.29 (-0.44, 1.02)	-0.06 (-0.21, 0.08)	-0.002 (-0.21, 0.20)	-0.18 (-0.55, 0.20)
**T_4 / Free T_4**	**-0.15 (-0.28, -0.03)** *	**0.37 (0.13, 0.62)** **	-0.04 (-0.09, 0.01)	**0.08 (0.01, 0.15)** *	-0.003 (-0.13, 0.12)
**T_3 / TBG**	0.07 (-0.04, 0.19)	-0.19 (-0.42, 0.04)	0.004 (-0.04, 0.05)	-0.03 (-0.09, 0.04)	0.01 (-0.11, 0.14)
**T_4 / TBG**	0.07 (-0.06, 0.20)	-0.10 (-0.35, 0.15)	0.001 (-0.05, 0.05)	-0.01 (-0.09, 0.06)	0.04 (-0.10, 0.17)
**Free T_4 / TBG**	0.14 (-0.01, 0.29)	**-0.35 (-0.64,-0.07)** *	0.02 (-0.04, 0.08)	**-0.09(-0.18,-0.01)** *	0.07 (-0.09, 0.23)

[a] adjusted for age and creatinine; * $p < 0.05$, ** $p < 0.01$, *** $p < 0.001$.

Table S5. Multiple linear regression [a] in concentrations of thyroid hormones in association with unit change in oxidative/ nitrosative stress biomarkers concentrations in visit 3 (N=58).

**Thyroid hormones/ oxidative/ nitrosative stress biomarkers**	**8-OHdG**	**8-NO_2Gua**	**HNE-MA**	**8-isoPGF$_{2\alpha}$**	**MDA**
	β (95%CI)	β (95%CI)	β (95%CI)	β (95%CI)	β (95%CI)
**TSH**	-0.14 (-0.73, 0.44)	-0.52 (-2.30, 1.25)	-0.05 (-0.27, 0.16)	-0.13 (-0.38, 0.11)	0.25 (-0.36, 0.87)
**T_3**	-0.01 (-0.18, 0.15)	-0.01 (-0.51, 0.49)	0.04 (-0.03, 0.10)	-0.03 (-0.10, 0.04)	-0.10 (-0.27, 0.08)
**T_4**	-0.04 (-0.14, 0.06)	0.20 (-0.11, 0.50)	0.02 (-0.02, 0.06)	-0.01 (-0.06, 0.03)	-0.04 (-0.15, 0.06)
**Free T_4**	-0.03 (-0.12, 0.06)	0.003 (-0.28, 0.28)	0.01 (-0.03, 0.04)	0.02 (-0.02, 0.06)	**-0.11 (-0.21, -0.01)** *
**TBG**	-0.03 (-0.18, 0.12)	0.07 (-0.38, 0.51)	0.04 (-0.02, 0.09)	-0.05 (-0.11, 0.01)	0.13 (-0.03, 0.28)
**T_3 / TSH**	0.13 (-0.49, 0.75)	0.51 (-1.35, 2.38)	0.09 (-0.14, 0.32)	0.10 (-0.16, 0.36)	-0.35 (-1.00, 0.29)
**T_3 / T_4**	0.03 (-0.13, 0.19)	-0.21 (-0.69, 0.27)	0.02 (-0.04, 0.08)	-0.02 (-0.08, 0.05)	-0.05 (-0.22, 0.11)
**TSH / T_4**	-0.11 (-0.72, 0.51)	-0.72 (-2.56, 1.12)	-0.07 (-0.30, 0.15)	-0.12 (-0.37, 0.14)	0.30 (-0.34, 0.93)
**TSH / Free T_4**	-0.11 (-0.72, 0.49)	-0.53 (-2.35, 1.30)	-0.06 (-0.29, 0.16)	-0.15 (-0.40, 0.10)	0.36 (-0.27, 1.00)
**T_4 / Free T_4**	-0.01 (-0.11, 0.09)	0.20 (-0.11, 0.51)	0.01 (-0.03, 0.05)	-0.03 (-0.08, 0.01)	0.07 (-0.04, 0.18)
**T_3 / TBG**	0.02 (-0.16, 0.19)	-0.08 (-0.60, 0.45)	-0.001 (-0.07, 0.06)	0.02 (-0.05, 0.09)	-0.22 (-0.40, -0.04)
**T_4 / TBG**	-0.01 (-0.14, 0.12)	0.13 (-0.26, 0.52)	-0.02 (-0.07, 0.03)	0.03 (-0.02, 0.09)	-0.17 (-0.31, -0.03)
**Free T_4 / TBG**	-0.001 (-0.18, 0.18)	-0.06 (-0.60, 0.47)	-0.03 (-0.10, 0.04)	0.07 (-0.01, 0.14)	**-0.24 (-0.42, -0.05)** *

[a] adjusted for age and creatinine; * $p < 0.05$, ** $p < 0.01$, *** $p < 0.001$.
